# Electrocardiographic and electrophysiological characteristics of idiopathic ventricular arrhythmias with acute successful ablation at the superior portion of the mitral annulus

**DOI:** 10.1186/s12872-021-02205-0

**Published:** 2021-08-18

**Authors:** Chengye Di, Konstantinos P. Letsas, Peng Gao, Qun Wang, Yanxi Wu, Wenhua Lin

**Affiliations:** 1 Cardiac Electrophysiology Unit, First Department of Cardiology, TEDA International Cardiovascular Hospital, 3rd Street, Tianjin Economic-Technological Development Area, Tianjin, 300457 China; 2 College of Clinical Cardiology, Tianjin Medical University, Tianjin, China; 3 Cardiovascular Institute, Tianjin University, Tianjin, China; 4 Second Department of Cardiology, Laboratory of Cardiac Electrophysiology, Evangelismos General Hospital of Athens, 10676 Athens, Greece

**Keywords:** Catheter ablation, Electrophysiology mapping, Electrograms, Ventricular arrhythmia, Mitral annular, Superior portion

## Abstract

**Background:**

We sought to identify the electrocardiographic and electrophysiological characteristics of ventricular arrhythmias (VAs), including idiopathic ventricular tachycardia (VT) and premature ventricular contractions (PVCs), with acute successful radiofrequency catheter ablation (RFCA) at the superior portion of the mitral annulus (SP-MA).

**Methods and results:**

Among 437 consecutive patients who presented with VAs for RFCA, twenty-six patients with acute successful RFCA at the SP-MA were included in this study. The ratio of the amplitude of the first positive peak (if present) versus the nadir in the unipolar electrogram (EGM) was 0.00–0.03 (0.00) at the acute successful RFCA site. The time interval between the QRS onset and the maximum descending slope (D-Max) in the unipolar EGM (QRS-Uni) was 18.8 ± 13.6 ms. With bipolar mapping, the ventricular QRS (V-QRS) interval was 3.75–17.3 (11) ms, 6 (23.1%) patients showed the earliest V-QRS interval of 0 ms, and the other 20 patients (76.9%) showed a V-QRS interval of 10–54 ms. The RFCA start-to-effect time was 14.1 ± 7.2 s in 23 patients (88.5%). In the remaining 3 patients (11.5%), the mean duration of successful RFCA was not well defined due to the infrequent nature of clinical VAs during RFCA. Early (within 3 days) and late (1-year) recurrence rates were 23.1% (6 patients) and 26.9% (7 patients), respectively. VAs disappeared 3 days later due to delayed RFCA efficacy in 2 patients (7.7%). No complications occurred during the RFCA procedure or the one-year follow-up.

**Conclusions:**

SP-MA VAs are a rare but distinct subgroup of VAs. Bipolar and unipolar EGM features can help to determine the optimal RFCA site, and the QRS-Uni interval may serve as a marker that could be used to guide RFCA.

## Background

Most idiopathic ventricular arrhythmias (VAs), including idiopathic ventricular tachycardia (VT) and premature ventricular contractions (PVCs), is either the right ventricular outflow tract (RVOT) or left ventricular outflow tract (LVOT). However, some idiopathic VAs may arise from various anatomical sites, including the right or left ventricular epicardial site, the aortic sinus cusp (ASC), aortomitral continuity (AMC), near the superior portion of the mitral annulus (SP-MA), and other sites [1]. Radiofrequency catheter ablation (RFCA) has emerged as a treatment for VAs, with a fairly high success rate. Recently, several cases of VAs have been reported to be successfully ablated at the mitral annulus (MA) and the SP-MA in close proximity to the AMC [2, 3]. The QRS morphology on electrocardiogram (ECG) of the VAs originating from the ASC, AMC, left ventricular summit area or SP-MA can mimic each other because of their anatomical vicinity [4]. However, little is known about the prevalence of SP-MA VAs, their ECG and electrophysiological (EP) characteristics, the efficacy of RFCA in treating SP-MA VAs and follow-up findings of SP-MA VAs. This study was performed to clarify these points.

## Methods

### Study participants

In this observational study, among 437 consecutive patients who presented with VAs for RFCA, including idiopathic VT and PVCs, between July 2010 and August 2018, 26 (5.9%) patients were found to have an acute successful RFCA site at the SP-MA during the index procedure. None of these patients exhibited significant coronary artery disease on coronary angiography or CT coronary angiography findings or any structural heart disease. Beta-blocker treatment or at least one antiarrhythmic drug therapy failed in these patients. Monomorphic nonsustained VT (defined as three or more consecutive PVCs) was present in 3 patients, and monomorphic PVCs were seen in the remaining 23 patients. All patients were in normal sinus rhythm (SR) before RFCA. Twelve-lead ECG and 24-h ambulatory Holter ECG were carried out at least once before RFCA. Demographic and clinical data, including patient age, sex, height, weight, biochemical blood examination results, echocardiographic parameters and clinical arrhythmias, were collected prior to the index procedure.

### ECG analysis

Twelve-lead ECGs were recorded utilizing the Libang Electrical system (Libang ECG recording, Libang Medical, Shenzhen, China). The ECGs were analyzed at a paper speed of 25 mm/s, and the signals were amplified at 10 mm/mV. The following parameters of VAs were analyzed: (1) the QRS amplitude in the inferior leads; (2) the QRS width; (3) the maximum deflection index (MDI), defined as the duration from the earliest activation to the peak of the largest amplitude deflection divided by the total QRS duration, measured in the precordial leads; (4) the peak deflection index (PDI), defined as the duration from the earliest activation to the peak of the largest amplitude deflection divided by the total QRS duration, measured in the inferior leads; (5) the S-wave in lead V_6_; and (6) inferior lead notching. All parameters were measured with electronic calipers by 3 experienced investigators blinded to the site of origin. We adopted the mean values of these measurements as the data. If the interobserver difference was more than 5 ms, the final decision was made by a joint meeting of the observers.

### Preparation before RFCA

All antiarrhythmic drugs were discontinued at least 5 half-lives before the EP study. Intracardiac tracings were recorded utilizing a Prucka CardioLab™ electrophysiology system (General Electric Health Care System, Inc., Milwaukee, WI, USA). If clinical VAs did not occur spontaneously and were not induced at baseline, intravenous isoproterenol (0.5–2.0 g/min) was administered to patients to induce clinical VAs. A 7.5-French, 3.5-mm-tip, irrigated ablation catheter (NaviStar ThermoCool, Biosense Webster, Diamond Bar, CA, USA) was then introduced into the left ventricle (LV) using a transaortic or transseptal approach. Also, intravenous heparin was administered to maintain an activated clotting time of 250–300 s.

### Electrogram (EGM) collection and analysis

**Fig. 1:**
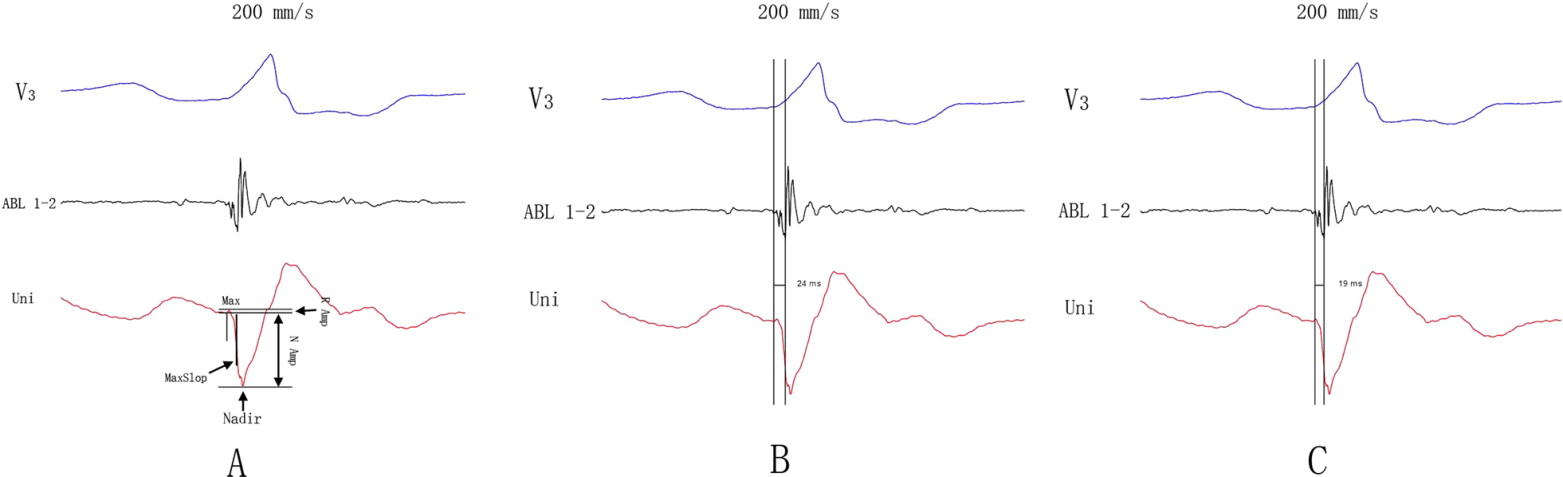
Illustration of key quantitative features on a sample bipolar and unipolar electrogram (EGM). R_amp, the amplitude of the first positive peak of the unipolar EGM; N_amp, the amplitude of the nadir of the unipolar EGM; MaxSlope, the maximum descending slope of the Q-wave; and maximum descending slope (D-Max), the time interval between the initial descent point to the MaxSlope. **a** R-ratio, the amplitude of the first positive peak relative to that of the nadir. **b** QRS-Uni interval, calculated from the QRS onset to the D-Max of the unipolar EGM. **c** V-QRS interval, calculated from the start of the bipolar ventricular EGM to the QRS onset

During an episode of clinical VA, activation mapping was performed. A minimum of three arrhythmic beats were recorded at the mapping site. Unipolar EGMs were recorded from the distal (D) electrode of the mapping catheter and filtered at 0.5–100 Hz. Bipolar EGMs were recorded from the distal (D-2) electrode pairs of the mapping catheter and filtered at 30–500 Hz. All EGM and twelve-lead ECG data were stored on the multichannel mapping system for offline analysis with a paper speed of 100 mm/s [5]. As shown in Fig. 1, the morphological features could be used to determine the R-ratio, which was derived from the unipolar EGM data as the amplitude of the first positive peak relative to that of the nadir (R-ratio). If no positive peak was present, then the R-ratio was 0. The QRS-Uni interval was calculated from the QRS onset to the maximum descending slope (D-Max) in the unipolar EGM. The V-QRS interval was calculated from the start of bipolar ventricular EGM to QRS onset. All parameters were measured with electronic calipers by 3 experienced investigators blinded to the site of origin. We adopted the mean values of these measurements as the data. If the interobserver difference was more than 5 ms, the final decision was made by a joint meeting of the observers.

### Pace mapping

Pace mapping was also performed at the earliest activation site using the distal bipolar electrodes at a coupling interval of the VA interval and a stimulus amplitude 1 mA greater than the late diastolic threshold (up to a maximum output of 10 mA and pulse width of 2.0 ms). If present, a perfect pace-mapping match (12/12 leads) was defined as indicating the site of origin; otherwise, the activation mapping result was only used for guiding RFCA.

### RFCA

RFCA was performed at the site where the earliest V-QRS, shortest QRS-Uni interval, or perfect pace-mapping match on ECG were recorded. RFCA was performed using the power-control mode at a maximum power of 35 to 40 W and temperature of 43 °C with irrigation mode at a flow rate of 17 mL/min. If the VAs were not eliminated within 30 s after energy delivery, energy application was terminated, and the RFCA site was tagged on the map as an unsuccessful site. If the VAs were abolished within 30 s, energy application was continued for a total of 300–500 s, and the site was tagged on the map as a successful site.

### Definition of acute successful RFCA

Acute successful RFCA was defined according to the following criteria: absence of spontaneous or induced clinical VAs, both in the absence and presence of isoproterenol infusion after RFCA with observation lasting 30 min to 1 h.

### Definition of SP-MA origin

**Fig. 2:**
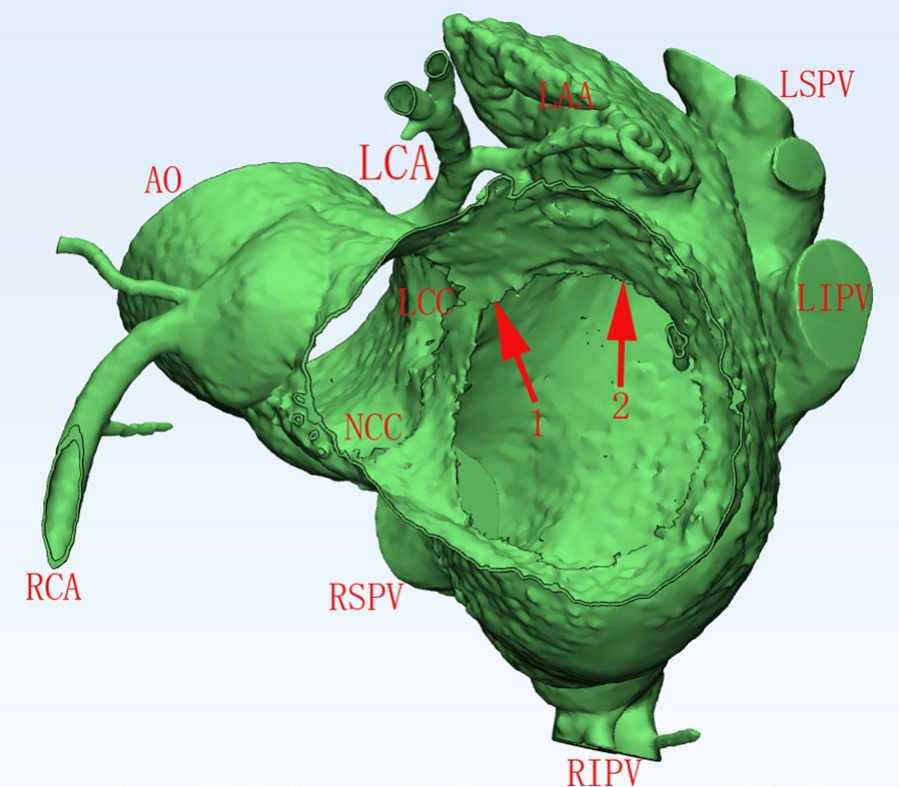
Graphical illustration of the SP-MA location in the nonstandard LAO view. Arrow 1 indicates the septal boundary of the SP-MA, which is the anatomic location of the AMC. Arrow 2 indicates the free wall boundary of the SP-MA. VAs originating from this location showed a monophasic R-wave and no S-wave in the inferior leads during VAs

We defined each SP-MA location as follows: (1) the catheter tip demonstrated that the characteristic SP-MA location and motion when viewed on the left and right anterior oblique fluoroscopic views at the successful RFCA site; (2) the catheter tip could be curved and decurved freely on the left anterior oblique fluoroscopic view; (3) the ratio of atrial/ventricular (A/V) EGMs at the RFCA site was < 1, and the amplitudes of the A/V EGMs were > 0.08/0.5 mV at the RFCA site during the SR [3]; (4) the catheter tip was on the left side of the AMC based on the CARTO3 map; (5) acute successful VA elimination was achieved by RFCA energy delivery at the site; (6) the ECG data showed a monophasic R-wave and no S-wave in the inferior leads during VAs (Fig. 2).

### Observation after RFCA and at the one-year follow-up

The patients were monitored for at least 3 days in the hospital after undergoing RFCA, and twelve-lead ECG and 24-h ambulatory Holter ECG monitoring were carried out at least once every three months. The patients were followed in our outpatient arrhythmia clinic for one year after RFCA, and twelve-lead ECG and 24-h ambulatory Holter ECG monitoring were carried out at least once every three months. In addition, all patients were contacted by telephone at the time of manuscript preparation. Clinical success was defined as the patient being free of clinical VAs (symptomatic or asymptomatic) that were targeted during RFCA at the follow-up visit and showing at least an 80% reduction in VA burden documented on post-RFCA 24-h Holter ECG recording compared to the pre-RFCA VA burden.

### Statistical analysis

Continuous data are presented as the mean ± SD. Noncontinuous data are presented as the interquartile range and median in parentheses. A p value < 0.05 was considered to indicate statistical significance.

## Results

### Location and frequency of VAs

Of the 437 patients referred for RFCA of idiopathic VAs, twenty-six (5.9%) patients were found to have an acute successful RFCA site at the SP-MA. The other origin sites were registered as follows: RVOT, 41.2%; ASC, 30.2%; AMC, 7.3%; tricuspid annulus (TA), 6.9%; papillary muscle, 3.2%; lateral and posterior portion of the MA, 6.6%; great cardiac vein and anterior interventricular vein, 1.6%; and other sites, 3%. The mean age of the 26 patients with SP-MA VAs was 61 ± 9 years, and there were 14 males and 12 females. The clinical characteristics of these patients are summarized in Table 1.

**Table 1:** Characteristics of the study population

N = 26	
Age (years)	61 ± 9 (31–78)
Male sex	14/26 (53.8%)
BMI	25.2 ± 3.5
K	4.03 ± 0.37
Cr	67.7 ± 13.0
UA	319.2 ± 86.2
Glu	4.7–5.8 (5.2)*
LDL-C	3.08 ± 0.80
TC	3.9–5.4 (5.0)*
TG	1.60 ± 0.73
LA-D (mm)	34.0–40.0 (36.5)*
LVEDD (mm)	46.8–54.0 (48.5)*
LVEF (%) before ablation	56.0–65.0 (60.5)*
Clinical VAs	
Only PVC	23
PVC, nonsustained VT	3

Values are given as the mean ± SD (range) or n (%), unless otherwise indicated. BMI = body mass index

*The interquartile range and median in parentheses for non-normally distributed data, the same as in Tables 2 and 3

### ECG characteristics of VAs

All patients exhibited a right bundle branch block, inferior-axis QRS morphology and negative QRS complexes in augmented vector left (aVL) and augmented vector right (aVR) leads. As shown in Table 2, the R_I_, S_I_, R_II_, R_III_, Q_aVR_, Q_aVL_, and R_aVF_ amplitudes were 0.00–0.30 (0.08) ms, 0.50 ± 0.17 ms, 1.50 ± 0.47 ms, 1.66 ± 0.54 ms, 0.60–0.89 (0.80) ms, 0.94 ± 0.27 ms, and 1.51 ± 0.43 ms, respectively. Inferior lead notching was recorded in 14 patients (53.8%), and S-waves were present in lead V_6_ for 6 patients (23.1%). The duration of the QRS complexes during VA was 168 ± 18 ms. The MDI was 0.54 ± 0.07, and the PDI was 0.52 ± 0.11.

**Table 2:** QRS morphology during VAs on surface ECG

Pt. no.	QRS voltage in the inferior leads (mV)							QRS duration (ms)	MDI	PDI	S-waves in lead V_6_	Inferior lead notching
	R_I_ (mV)	S_I_ (mV)	R_II_ (mV)	R_III_ (mV)	Q_aVR_ (mV)	Q_aVL_ (mV)	R_aVF_ (mV)					
1	0.35	0.55	1.58	1.38	0.88	0.57	1.40	173	0.68	0.63	No	Yes
2	0.00	0.32	1.15	1.20	0.60	0.80	1.20	157	0.47	0.46	No	No
3	0.28	0.82	1.25	1.10	0.77	0.70	1.17	157	0.54	0.57	No	Yes
4	0.00	0.68	1.95	2.14	1.12	1.32	2.14	158	0.51	0.52	No	Yes
5	0.10	0.40	1.00	1.25	0.50	0.70	1.10	147	0.76	0.74	Yes	Yes
6	0.00	0.30	0.80	0.90	0.30	0.60	0.80	192	0.55	0.33	No	Yes
7	0.00	0.71	2.52	3.10	1.35	1.48	2.17	198	0.31	0.61	No	Yes
8	0.00	0.70	1.02	1.18	0.75	0.75	1.07	172	0.63	0.66	Yes	Yes
9	0.25	0.35	1.60	1.80	0.80	1.00	1.60	183	0.52	0.52	No	No
10	0.40	0.62	2.34	2.27	1.39	0.9	1.42	142	0.54	0.54	No	Yes
11	0.35	0.40	1.30	1.20	0.80	0.8	0.90	147	0.58	0.67	No	No
12	0.05	0.07	1.30	1.30	0.60	0.9	1.30	183	0.56	0.33	Yes	Yes
13	0.00	0.50	1.50	1.80	0.80	1.00	1.60	186	0.58	0.40	Yes	Yes
14	0.00	0.48	1.45	1.85	0.75	0.90	1.55	181	0.51	0.40	No	Yes
15	0.42	0.51	1.91	2.24	1.09	1.27	2.06	170	0.55	0.64	No	No
16	0.00	0.45	1.80	2.10	0.80	1.20	2.00	158	0.43	0.49	No	Yes
17	0.30	0.60	1.00	1.60	0.80	1.00	1.60	150	0.59	0.65	No	No
18	0.10	0.50	1.90	2.15	0.80	1.30	2.05	145	0.59	0.62	No	No
19	0.40	0.71	2.30	2.20	1.05	1.40	2.40	165	0.58	0.53	No	No
20	0.28	0.48	1.30	1.60	0.80	0.9	1.50	167	0.45	0.46	Yes	Yes
21	0.03	0.35	1.23	1.00	0.73	0.47	1.13	156	0.58	0.52	No	No
22	0.00	0.63	1.41	1.51	0.78	0.88	1.56	186	0.59	0.40	No	No
23	0.05	0.54	1.13	0.93	0.60	0.63	1.02	136	0.59	0.53	No	No
24	0.30	0.60	0.90	1.45	0.40	1.00	1.15	178	0.53	0.49	No	Yes
25	0.15	0.22	2.12	2.33	0.9	0.85	1.85	191	0.42	0.42	No	No
26	0	0.41	1.32	1.65	0.45	1.05	1.40	195	0.50	0.46	Yes	No
Mean ± SD or percent	0.00–0.30 (0.08)*	0.50 ± 0.17	1.50 ± 0.47	1.66 ± 0.54	0.60–0.89 (0.80)*	0.94 ± 0.27	1.51 ± 0.43	168 ± 18	0.54 ± 0.07	0.52 ± 0.11	23.1%	53.8%

Values are given as the mean ± SD (range) or percent (%), unless otherwise indicated. The maximum deflection index (MDI) was defined as the duration from the earliest activation to the peak of the largest amplitude deflection divided by the total QRS duration, measured in the precordial leads. (4) The peak deflection index (PDI) was defined as the duration from the earliest activation to the peak of the largest amplitude deflection divided by the total QRS duration, measured in the inferior leads

### Activation mapping of clinical VAs

**Fig. 3:**
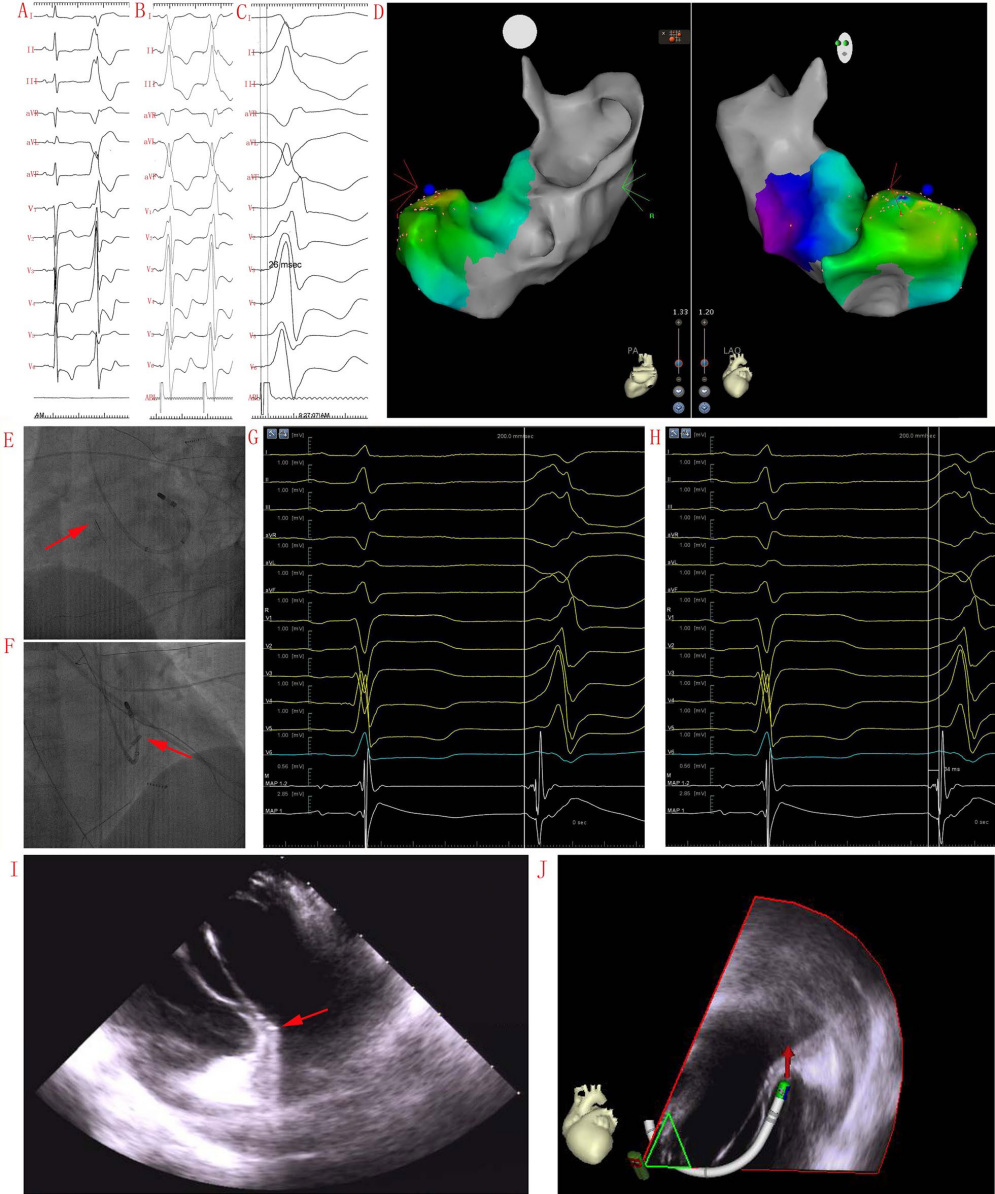
Patient 13 had premature ventricular contractions (PVCs) with acute successful RFCA at the superior portion of the mitral annulus (SP-MA). **a** Twelve-lead electrocardiographic (ECG) morphology of the QRS complex during sinus rhythm (SR) and PVCs (paper speed 25 mm/s). **b** Pace-mapping QRS complex morphology (paper speed 25 mm/s). **c** A stimulus-to-QRS interval of 26 ms with an excellent pace map was recorded at the acute successful RFCA site (paper speed 100 mm/s). **d** CARTO3 mapping indicates an acute successful RFCA site at the SP-MA. **e, f** Left and right anterior oblique fluoroscopic views indicate an acute successful RFCA site at the free wall of the SP-MA. The intra-cardiac echocardiography (ICE) catheter was advanced into the right ventricle to show the location of the RFCA catheter tip (arrow). **g** Earliest V-QRS interval of 0 ms for bipolar recording during PVCs (paper speed 100 mm/s) and an A/V ratio of 0.43 during SR. **h** QRS-Uni interval of 34 ms for unipolar recording during PVCs, with an R-ratio of 0.07 (paper speed 100 mm/s). **i**, **j** ICE showed that the ablation catheter tip was on the left side of the AMC (arrow)

Patients underwent coronary angiography and coronary venous angiography prior to mapping. Detailed mapping of the ASC, AMC, distal portion of the coronary venous, and SP-MA regions was performed in all 26 patients. No areas of abnormal endocardial voltage were seen in the SP-MA regions. In 437 patients treated by RFCA, acute successful RFCA at the SP-MA was achieved in 26 (5.9%) (in 25 patients with a transaortic approach and in 1 patient with a transseptal approach). As shown in Table 3, the A/V amplitude ratio at the RFCA site during SR was 0.23 ± 0.15. The QRS-Uni interval in the unipolar EGM was 18.8 ± 13.6 ms. The R-ratio in the unipolar EGM was 0.00–0.03 (0.00) at the successful RFCA site, and initial unipolar QS waves were recorded in 19 patients (73.1%). With bipolar mapping, the V-QRS interval was 3.75–17.3 (11) ms, 6 (23.1%) patients showed the earliest V-QRS interval of 0 ms, and the other 20 (76.9%) patients showed a V-QRS interval of 10–54 ms. A presystolic potential that preceded QRS onset was recorded during VA in 1 patient (3.8%). Figure 3d, g show the earliest V-QRS intervals of 0 ms and 31 ms, respectively, for bipolar recording during VAs.

**Table 3:** Electrophysiologic Characteristics and ablation result of AP-MA VAs

Pt. no.	Pre-potential	A/V ratio during sinus rhythm	R/S ratio during unipolar recording	QRS-Uni (ms)	Perfect pace match	Stimulus-to-QRS interval	V-QRS interval (ms)	RFCA start to effect (s)	Recurrence during 3 days of monitoring	Recurrence during 1 year of follow up
1	No	0.21	0.19	52	No	0	54	8.1	Yes	No
2	No	0.28	0.00	0	No	0	0	17.3	No	Yes
3	No	0.35	0.31	7	No	0	15	7.7	No	No
4	No	0.03	0.22	47	No	0	18	4.2	No	No
5	No	0.05	0.00	6	No	0	31	16.5	No	No
6	No	0.58	0.09	30	No	0	0	17.3	No	No
7	No	0.03	0.00	25	No	0	5	30.2	No	Yes
8	No	0.16	0.00	12	Yes	0	7	N/A	No	No
9	No	0.46	0.00	24	Yes	0	50	19.3	No	Yes
10	No	0.04	0.00	8	Yes	0	12	9.2	No	No
11	No	0.31	0.00	22	No	0	0	5.59	No	No
12	No	0.10	0.10	16	No	0	14	21.3	No	No
13	Yes	0.43	0.07	40	No	**26**	0	17.4	Yes	No
14	No	0.33	0.00	26	No	0	17	19.1	Yes	Yes
15	No	0.05	0.00	17	No	0	14	17.5	No	No
16	No	0.21	0.00	12	Yes	0	0	3.2	Yes	No
17	No	0.43	0.00	8	No	0	7	20.1	No	No
18	No	0.04	0.00	15	No	0	0	23.1	Yes	Yes
19	No	0.22	0.00	28	Yes	0	9	21.7	No	No
20	No	0.22	0.00	24	Yes	0	12	14.4	No	No
21	No	0.19	0.00	9	Yes	0	10	5.6	No	No
22	No	0.13	0.00	0	Yes	0	9	13.2	No	No
23	No	0.28	0.00	0	Yes	0	14	5.4	Yes	Yes
24	No	0.29	0.00	24	No	0	6	N/A	No	Yes
25	No	0.28	0.00	12	No	0	37	8.5	No	No
26	No	0.23	0.02	25	No	0	25	N/A	No	No
Mean ± SD or percent	3.8%	0.23 ± 0.15	0.00–0.03 (0.00)*	18.8 ± 13.6	34.6%		3.75–17.3 (11)*	14.1 ± 7.2^#^	23.1%	26.9%

Values are given as the mean ± SD (range) or percent (%), unless otherwise indicated. N/A: Time to effect were not available due to infrequently episode of VAs during ablation

^#^The RFCA start-to-effect time of 14.1 ± 7.2 s were for the 23 patients (88.5%), in the remaining 3 patients (11.5%), the mean duration of successful RFCA was not well determined due to infrequent nature of clinical VAs during ablation

### Pace mapping

The characteristics of the QRS morphology during pacing in 9 patients (34.6%) were almost identical to those of the VAs successfully ablated at the SP-MA. An interval of 26 ms between the pacing stimulus artifact and QRS onset was recorded in 1 patient (Fig. 3c).

### RFCA at the SP-MA

**Fig. 4:**
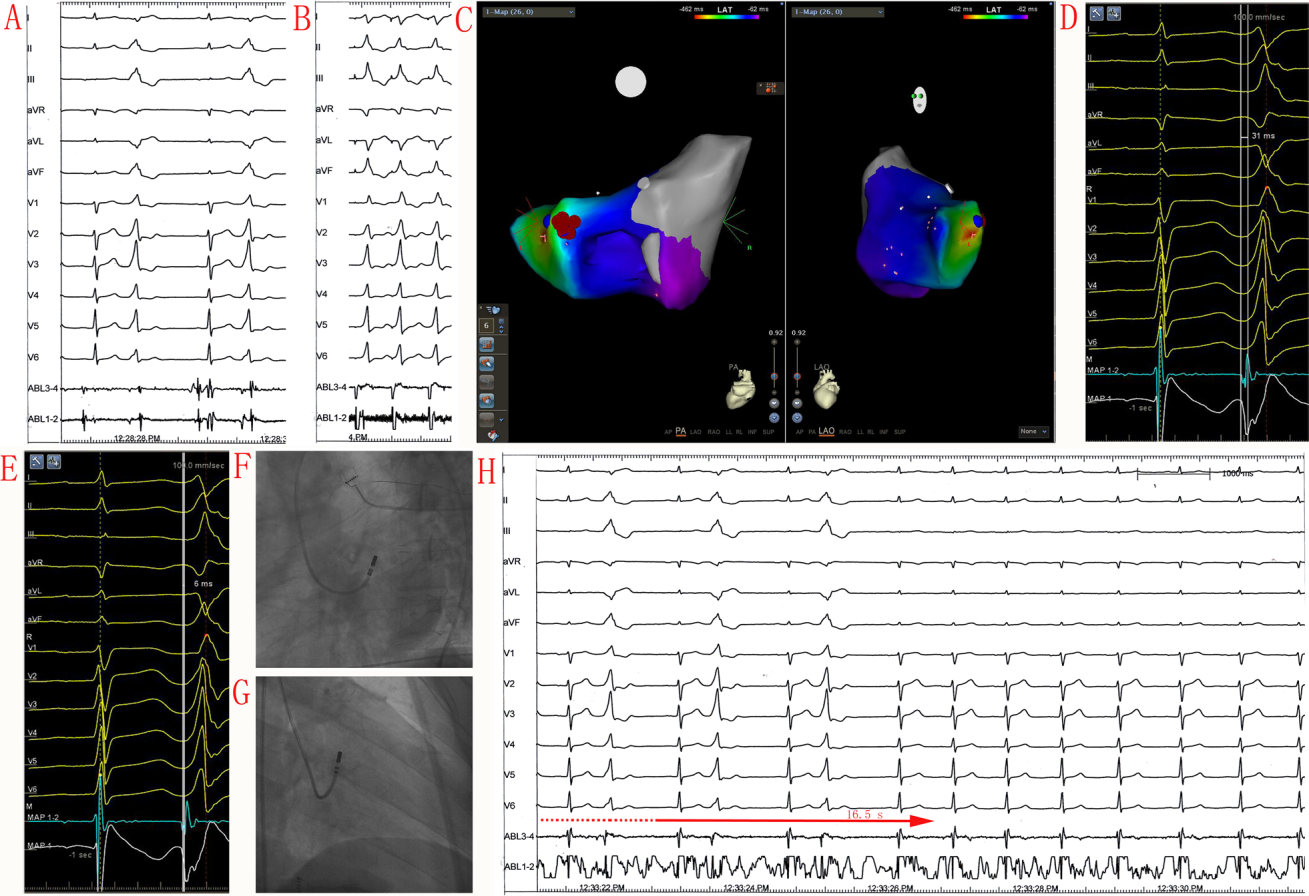
Patient No. 5 had PVCs with acute successful RFCA at the SP-MA. **a** Twelve-lead ECG morphology of the QRS complex during SR and PVCs (paper speed 25 mm/s). **b** Pace-mapping QRS complex morphology (paper speed 25 mm/s). **c** CARTO3 mapping indicates an acute successful RFCA site at the SP-MA. **d** Earliest V-QRS interval of 31 ms for bipolar recording during PVCs and an A/V ratio of 0.05 during SR (paper speed 100 mm/s). **g** QRS-Uni interval of 6 ms for unipolar recording during PVC and an R-ratio of 0 (paper speed 100 mm/s). **f, g** Left and right anterior oblique fluoroscopic views at the SP-MA. **h** Elimination of PVCs after RFCA application for 16.5 s

Complete elimination of VAs could be achieved by RFCA at the appropriate RFCA site, where the earliest V-QRS interval or shortest QRS-Uni interval was recorded at the SP-MA. The RFCA location for SP-MA VAs are shown in Figs. 3e, f and 4f, g (left and right anterior oblique fluoroscopic views). The RFCA start-to-effect time was 14.1 ± 7.2 s in 23 patients (88.5%). In the remaining 3 patients (11.5%), the mean duration of a successful RFCA procedure was not well determined due to the infrequent nature of clinical VAs during RFCA. No complications occurred during RFCA procedures.

### Observations after RFCA and at the 1-year follow-up

Clinical VAs could still be recorded in 6 patients (23.1%) during 3 days of in-hospital monitoring after RFCA. VAs disappeared in 7.7% (2/26) of patients 3 days later, which was 6 days after the RFCA procedure, due to delayed RFCA efficacy, and during the one-year follow-up period, 26.9% (7/26) of patients had clinical VA recurrence, and one patient underwent the procedure again. The final RFCA target was the same as the index procedure based on X-ray fluoroscopic views and CARTO3 mapping results.

## Discussion

The current study has four major findings. First, the frequency of SP-MA VAs confirmed by successful RFCA was 5.9% in 437 consecutive patients with idiopathic VAs in a single center. Second, the QRS-Uni interval in the unipolar EGM data was 18.8 ± 13.6 ms; thus, this interval could serve as a marker that could be used to guide RFCA and help reduce unnecessary RFCA applications. Third, the A/V amplitude ratio at the RFCA location of the SP-MA during SR was 0.23 ± 0.15. Fourth, RFCA was effective for the acute elimination of these SP-MA VAs.

The EP features of the SP-MA VAs include preferential conduction, multiple exits, ventricular prepotentials, and a long stimulus-to-QRS interval with pacing (Fig. 3c). These EP features and the anatomical complexity of SP-MA VAs may present challenges during the mapping and RFCA of VAs in this region [6]. In this study, only 6 (23.1%) patients exhibited the earliest bipolar activation preceeding the QRS onset of 0 ms at the acute successful RFCA site. This suggests that the VA origin may be immediately adjacent to the ventricular breakout, or it may be located at an epicardial or intramyocardial location that could not be recorded. A thorough understanding of the anatomical complexity of successful RFCA locations at the SP-MA has the potential to improve outcomes, decrease complication rates and minimize the need for repeat RFCA procedures. Usually, activation mapping is preferable to pace mapping. The effectiveness of pace mapping at the SP-MA in particular is reduced by a smaller and nonuniformly distributed myocardium that is available for electrical capture. This, in turn, requires higher electrical output, which can produce imperfect pace mapping results, even at the VA origin, owing to far-field capture. Furthermore, the accuracy of pace mapping may be diminished by preferential conduction at the SP-MA location. Our results indicate that VAs with acute successful RFCA at the SP-MA can be identified as one subgroup of VAs with distinctive ECG characteristics and that RFCA is effective for the acute elimination of these VAs.

The location of the RFCA target was identified in the posterior portion of the LV, and the myocardium at the RFCA target depolarized in the direction that toward these precordial electrodes. This occurrence could explain the precordial transition of R/S > 1 in lead V_1_ in most of these cases during VAs. The timing of excitation of the LV and right ventricle is considered to mainly affect QRS morphology and the duration in the inferior leads. When VAs originate near the AMC of the SP-MA, the ventricular septum and right ventricle would be depolarized earlier than VAs originating from the free wall of the SP-MA, which may result in the absence of notching in the inferior leads or the absence of an S-wave in lead V_6_.

As shown in Table 2, QRS complexes during VAs all showed a right bundle branch block pattern, S-waves were present in lead V_6_ in 6 patients (23.1%), and inferior lead notching was present in 14 patients (53.8%). Delta-wave-like onset of the precordial QRS complex during VAs may support the theory that VAs originated deep inside the subendocardium or epicardium at the SP-MA (Fig. 3a) [8, 9]. It is likely that some of these VAs might have overlapped with the VAs that originated from the AMC rather than with those of other origins [10, 11]. Li et al. reported that the R-wave amplitude in lead V_1_ was greater than that in lead V_2_ in 4 of 5 patients with VAs arising from the proximal portion of the anterior interventricular vein [12]. Kumagai et al. reported that SP-MA VAs had an Rs pattern in some precordial leads except for lead V_6_ [13]. A notched QRS pattern and a longer QRS duration in inferior leads may have indicated VAs originating from the free wall of the SP-MA in our study (Fig. 3a).

Although several studies have reported the successful ablation of VAs near the SP-MA, none have systemically reported the prevalence, ECG and EP characteristics of VAs originating from the SP-MA and analyzed the follow-up results in as many patients as were evaluated in this study [2, 3]. The SP-MA is close to the posterior portion of the RVOT, the LV summit myocardium near the left sinus cusp, and the subvalvular portion of the LVOT; these areas are reported as foci of VAs originating from the LVOT/RVOT [6, 7]. The close anatomic proximity suggests that different forms of VAs could likely originate from one single origin or from the activation of alternative pathways between the VA focus and an exit point [4].

Kumagai et al. reported that VAs arising from the MA had delta wave-like QRS morphologies and were sensitive to isoproterenol and ATP but insensitive to programmed pacing or burst pacing [14]. These findings support a mechanism of triggered activity rather than a reentrant mechanism. Anderson et al. identified AV ring-specialized tissue at the SP-MA that differed in histological and histochemical characteristics [15]. We hypothesized that the target site around the SP-MA would reflect the activation of a segment of AV ring-specialized tissue that is poorly coupled with the surrounding ventricular myocardium.

The conventional strategy to guide RFCA in the treatment of VAs is based on activation mapping during bipolar recording and the presence of QS waves during unipolar recording [16–18]. In this study, complete initial unipolar QS waves were recorded in 18 patients (69.2%). The initial QS waves suggested that the ablation catheter tip was just at the VA origin or near the ventricular breakout point without conduction delay. The QRS-Uni interval may be related to how fast the wave front was conducted from the recording location of the mapping catheter to the breakout point. Thus, this interval could serve as a marker that could be used to guide RFCA, help reduce unnecessary RFCA applications and predict long-term success rates. As shown in Table 3, with bipolar mapping, the V-QRS interval was 3.75–17.3 (11) ms, 6 (23.1%) patients showed the earliest V-QRS interval of 0 ms, and the other 20 patients (76.9%) showed a V-QRS interval of 10–54 ms. Excellent leading V-potentials from the successful RFCA site could not be identified in more than half of these cases, indicating an intramural or epicardial origin of these VAs.

Successful catheter RFCA was achieved underneath the SP-MA using a transaortic approach in 25 patients (96.2%); in one patient, RFCA could be achieved only by using a transseptal approach due to the anatomical complexity of the SP-MA. The RFCA start-to-effect time was 14.1 ± 7.2 s in 23 patients (88.5%). In the remaining 3 patients (11.5%), the mean duration of successful RFCA was not well determined due to the infrequent nature of clinical VAs during RFCA. Clinical VAs could still be recorded in 6 patients (23.1%) during 3 days of in-hospital monitoring after RFCA. Also, 7.7% (2/26) of VAs disappeared 3 days later due to delayed RFCA efficacy, which indicated that waiting for delayed RFCA efficacy is also a reasonable choice in cases of VAs arising from the region of the SP-MA [19].

Epicardial RFCA via the transpericardial approach is not suitable for the treatment of idiopathic VAs originating from the LV summit because of anatomic barriers, such as close proximity to the coronary arteries, thick epicardial fat pads, and/or unreachable anatomical limitations [20]. The sites of VAs originating from the LV summit area are roughly predicted by the mapping information from the corresponding endocardial SP-MA, ASC, AMC or other areas based on the assumption that the conduction properties in these regions are normal. Komatsu et al. reported that mapping the LV summit by using a microcatheter is feasible and helpful for identifying the site of origin. In his study, it was not possible to advance a standard irrigated-tip ablation catheter to the LV summit area, and ablation was attempted in adjacent structures [21]. Therefore, RFCA at nearby sites, such as the SP-MA, is one of the alternative ablation techniques to treat these VAs in our study. Recognition of these possibilities is helpful for RFCA of such VAs originating from the SP-MA.

There are limitations to this retrospective study. First, the exact origins of VAs may be adjacent origins, including the anterior interventricular vein, great cardiac vein and other epicardial locations. However, these locations cannot be confirmed or ablated because the ideal target sites are not reachable due to anatomical limitations. Second, intracardiac echocardiography was not used to confirm the RFCA target. Therefore, the exact origins of VAs could not be ascertained. Third, the target site might not have precisely coincided with the VA origin, although we believe that the target site was very close. Fourth, while the potential preceding the QRS complexes was recorded at successful RFCA sites, its significance and genesis were not determined. Further evaluation with a prospective study design is needed to assess the reproducibility of RFCA at SP-MA VAs.

## Conclusions

VAs with acute successful RFCA at the SP-MA are a rare but identifiable subgroup of VAs with distinctive EP and ECG characteristics. RFCA is effective for the acute elimination of these VAs. Advanced knowledge of the SP-MA anatomy, ECG features and EP features may be useful in planning and/or facilitating RFCA procedures. Aside from the bipolar V-QRS interval, the QRS-Uni interval could serve as a marker that could be used to guide RFCA and help reduce unnecessary RFCA applications.

## Data Availability

The datasets used and/or analyzed during the current study are identified and available from the corresponding author on reasonable request.

## Abbreviations

VAs: Ventricular arrhythmias
VT: Ventricular tachycardia
PVC: Premature ventricular contraction
RFCA: Radiofrequency catheter ablation
SP-MA: Superior portion of the mitral annulus
EGM: Electrogram
RVOT: Right ventricular outflow tract
LVOT: Left ventricular outflow tract
ASC: Aortic sinus cusp
AMC: Aortomitral continuity
ECG: Electrocardiogram
EP: Electrophysiological
SR: Sinus rhythm
MDI: Maximum deflection index
PDI: Peak deflection index
LV: Left ventricle
TA: Tricuspid annulus
RCA: Right coronary artery
LCA: Left coronary artery
AO: Aorta
LCC: Left coronary sinus
RCC: Right coronary sinus
LAA: Left atrial appendage
LSPV: Left superior pulmonary vein
RSPV: Right superior pulmonary vein
LAA: Left superior pulmonary vein
